# The Panel Subgroup (PSG) Method: A Valuable Method for Effectively Engaging Panels in the Development of Practice Guidelines

**DOI:** 10.1002/gin2.70042

**Published:** 2025-09-01

**Authors:** Nofisat Ismaila, Brittany E. Harvey, Kaitlin Einhaus, Lawrence Mbuagbaw, Jinhui Ma, Lehana Thabane

**Affiliations:** ^1^ Department of Health Research Methods Evidence, and Impact McMaster University Hamilton Ontario Canada; ^2^ American Society of Clinical Oncology Alexandria Virginia USA; ^3^ Department of Anesthesia McMaster University Hamilton Ontario Canada; ^4^ Department of Pediatrics McMaster University Hamilton Ontario Canada; ^5^ Biostatistics Unit, Father Sean O'Sullivan Research Centre, St Joseph's Healthcare Hamilton Ontario Canada; ^6^ Centre for Development of Best Practices in Health (CDBPH), Yaoundé Central Hospital Yaoundé Cameroon; ^7^ Department of Global Health, Division of Epidemiology and Biostatistics Stellenbosch University Cape Town South Africa; ^8^ St. Joseph's Healthcare‐Hamilton Hamilton Ontario Canada; ^9^ Faculty of Health Sciences, University of Johannesburg Johannesburg South Africa

**Keywords:** expert panel, guideline, panel subgroup, retrospective review

## Abstract

**Background:**

Clinical practice guidelines rely on expert panels to review evidence and develop recommendations. The traditional approach of engaging experts often relies on periodic full panel meetings which can lead to slow progress and variability in panel engagement. To address these issues, the ASCO introduced the Panel Subgroup (PSG) method, dividing panels into smaller working groups to enhance engagement, distribute workload, and accelerate guideline development.

**Objective:**

To evaluate the PSG method on guideline development timelines, its uptake, perceived benefits, challenges, and lessons learned from the perspective of ASCO guideline methodologists.

**Methods:**

This study had two phases. Phase 1 was a retrospective review of ASCO guideline administrative data (2017–2024) comparing guidelines developed using the traditional or PSG method. Guidelines were included if they were de novo, had over 50 included studies, and complete administrative records. Data extracted included number of panelists, research questions, studies reviewed, subgroups (for PSG), and development time. Phase 2 involved qualitative interviews with methodologists who used the PSG method to explore experiences, benefits, and challenges.

**Results:**

Sixteen guidelines met the inclusion criteria: seven used the traditional method and nine used the PSG method. The average development time was 13 months (SD, 2.41) for the PSG method compared to traditional (23 months; SD, 6.30). PSG uptake among methodologists was 75% (6/8). Reported benefits included improved expert engagement, deeper evidence analysis, better collaboration, and shared authorship. Challenges included greater time demands, increased coordination, writing inconsistencies, and the need for full panel buy‐in.

**Conclusion:**

The PSG method offers a promising structure to improve engagement and streamline productivity. While not without challenges, it may be particularly useful for large panels and complex guidelines when applied thoughtfully.

## Summary

- **What Is Already Known on This Topic:**
  ∘ Clinical practice guidelines often involve large multidisciplinary expert panels that are prone to challenges of maintaining engagement and efficiency.
  ∘ Traditional approaches to guideline development often overburden co‐chairs and select members while leaving other panelists disengaged for extended periods.
  ∘ There are limited empirical evaluations of approaches to maintain expert panel engagement on guideline panels.
- **What This Study Adds:**
  ∘ The panel subgroup (PSG) method, which organizes expert panels into smaller working subgroups to streamline workflows, distribute responsibilities, and promote engagement of guideline panels.
  ∘ Evidence from ASCO's experience in using the PSG method shows that it enhances workflow efficiency by reducing guideline development delays and promoting steady progress through frequent subgroup meetings and communication.
  ∘ Methodologists observed improved panelist engagement and collaboration, as smaller groups enabled more thorough discussions and fostered professional connections among members.
  ∘ Challenges identified include harmonizing diverse writing styles, addressing duplicative efforts, ensuring subgroup expertise, and importance of early buy‐in from panelists.
  ∘ This study emphasizes critical skills required for successful implementation, including strong organizational, communication, and time‐management abilities.
- **Key Takeaways:**
  ∘ The PSG method provides a promising framework for improving guideline development by fostering collaboration, increasing efficiency, and promoting sustained engagement.

## 1 Introduction

Clinical practice guidelines play a crucial role in standardizing care by providing evidence‐based recommendations to clinicians and patients [1]. The success of these guidelines relies significantly on expert panel members, who voluntarily contribute their time and expertise to reviewing evidence, engaging in thoughtful deliberation, and reaching consensus on recommendations [2, 3, 4]. Guideline development requires the coordination of multidisciplinary experts through several stages, including protocol planning, systematic literature review, evidence synthesis, recommendation drafting, and manuscript preparation [5].

In the traditional guideline development method, expert panels are engaged through multiple full group sessions to collaborate on guideline development. However, this approach often presents challenges: (1) recommendations may be disproportionately influenced by dominant voices on the panel, (2) diverse and sometimes large panels bring a range of perspectives that can be difficult to integrate, (3) the extensive volume of evidence and research questions may overwhelm the process, (4) panelists may experience reduced engagement or motivation, and (5) the process can be highly time‐intensive [6, 7].

**Figure 1:**
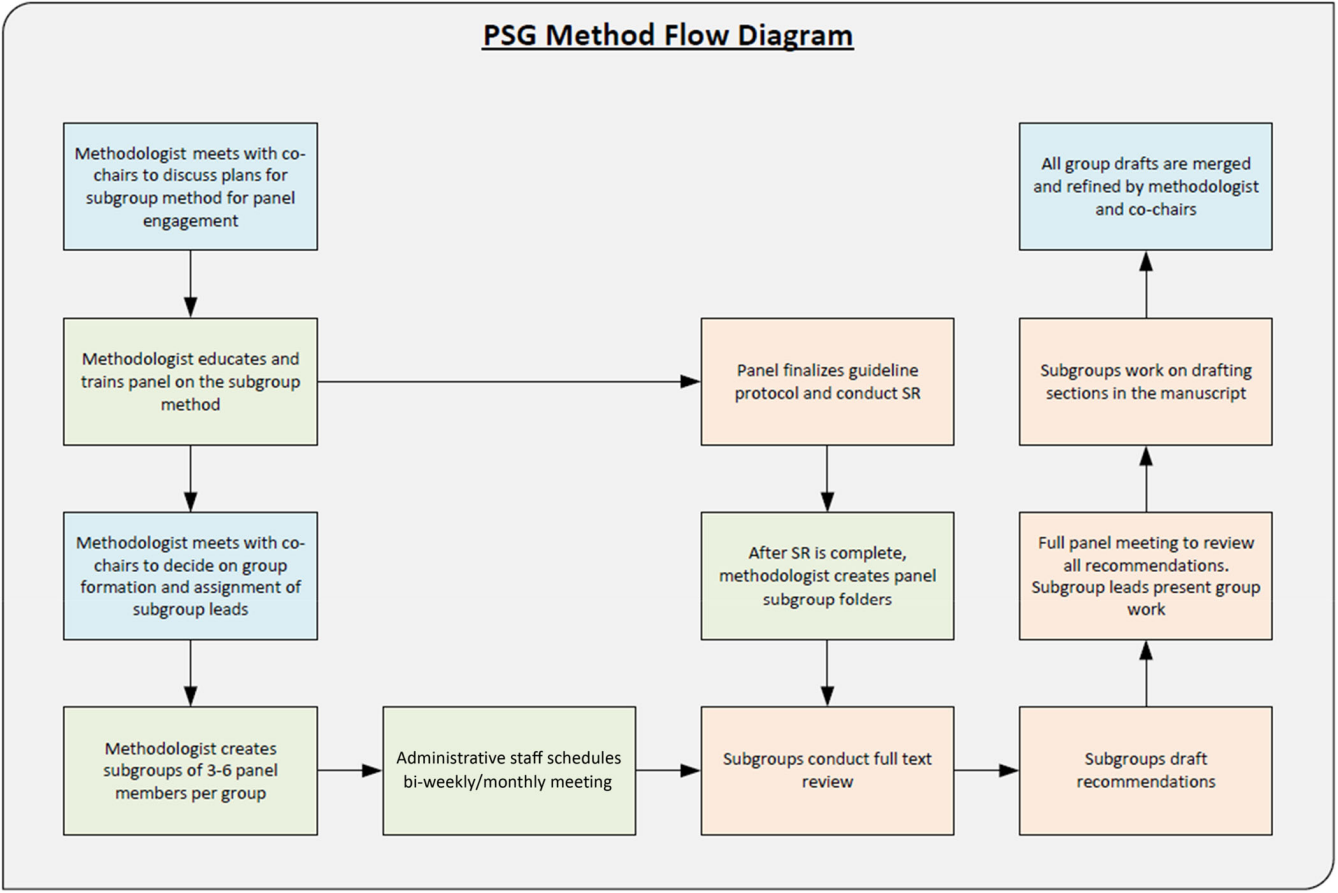
PSG method flow diagram. PSG, panel subgroup; SR, systematic review.

The American Society of Clinical Oncology (ASCO) is a leading US organization that develops evidence‐based clinical practice guidelines for oncology, offering recommendations to clinicians and patients [8]. ASCO maintains a comprehensive library of over 200 guidelines across 15 clinical and topical areas, developing approximately 25 new guidelines annually through expert volunteer panels [9]. Since its inception, the ASCO guideline program primarily employed the traditional method of engaging expert panel members. However, in 2017, a new approach known as the Panel Subgroup (PSG) method (Figure 1) was introduced in a subset of guideline projects. This shift was driven by the increasing need to manage larger panels assembled to address complex clinical questions, need to expedite the development process, which in turn, allows for more guidelines to be developed, as well as feedback from a volunteer survey that indicated declining engagement among expert panelists.

### 1.1 The PSG Method

In this approach, the expert panel is divided into smaller working groups within the panel to distribute tasks evenly based on their expertise and relevance to guideline questions. Each working group typically consists of three to six members, with the exact number determined by the overall size of the guideline panel and the number and complexity of the clinical questions being addressed. One member from each group is designated as the group lead, responsible for coordinating the subgroup's activities and serving as the primary point of contact with the methodologist and co‐chairs. These working groups meet regularly, coordinated by an administrative staff, typically on a biweekly or monthly basis, depending on the project timeline. A methodologist is present at each meeting to provide methodological guidance, support, and ensure consistency across groups. During these meetings, members collaboratively review the relevant body of evidence, interpret the findings, and draft preliminary recommendations using the GRADE Evidence‐to‐Decision (EtD) framework. They are also responsible for drafting assigned sections of the guideline manuscript, including evidence interpretation and rationale statements. Each working group, led by the group lead, presents its preliminary recommendations along with supporting evidence summaries and rationale during a full panel, meeting where all proposed recommendations are reviewed and finalized by the entire panel. If disagreements arise, the panel engages in structured deliberation facilitated by the methodologist until consensus is reached. This structure fosters, transparency, rigor, shared ownership of the work, allows for more focused discussion on specific topics, and distributes the workload more evenly across the panel.

#### 1.1.1 Administrative Support and Coordination

To support the operational demands of the PSG method, ASCO assigns dedicated administrative staff to each guideline project. These staff members are responsible for scheduling subgroup meetings, and tracking overall project progress. This administrative support ensures coordination across subgroups and enables the methodologist to focus on providing methodological guidance. The collaboration between methodologists and administrative staff has proven essential to maintaining a steady workflow and timely development of the guidelines.

The use of the PSG method at ASCO was not mandated; rather, methodologists determined its applicability based on the specific needs of each guideline and their own comfort level with the approach.

This study aims to evaluate the PSG method on guideline development timelines. The uptake, perceived benefits, challenges, and lessons learned using this approach among guideline methodologists at ASCO were also assessed.

## 2 Methods

This study was conducted in two phases. The first phase involved a retrospective review of administrative data on guideline development milestones. ASCO's guideline team maintains a tracking database for all guidelines, which contains essential data such as guideline codes, status (new, update, or archived), names of co‐chairs and panel members, assigned staff, and key milestone dates (e.g., initiation, panel invitation, protocol approval, systematic review, guideline approval, and publication). This database is regularly updated by administrative staff and the methodologists assigned to each guideline.

The following eligibility criteria were used to identify guidelines from this database:

### 2.1 Inclusion Criteria

- Guidelines published between 2017 and 2024.
- De novo (new) guidelines.
- Inclusion of more than 50 studies in the systematic review component of the guidelines.
- Complete guideline administrative data available, from protocol approval to final committee approval.

### 2.2 Exclusion Criteria

- Guideline updates.
- Guideline endorsements.
- Clinical opinion‐only guidelines.
- Living guidelines.

These criteria guided the selection of guidelines from the tracking database, allowing for a focused review of guideline processes and outcomes specific to this new panel engagement approach.

To better understand the perceived benefits, challenges, and uptake of the PSG method, a qualitative approach was used in the second phase of the study. ASCO guideline methodologists who implemented the PSG method were asked to provide insights through a structured set of questions via either email correspondence or phone interview. The inquiry covered key areas of their workflow, panel engagement, skill requirements, and reflections on the practical challenges and benefits of the method. The following questions were included in the discussion guide:

1. Frequency of PSG Method Use
*Objective: To contextualize responses by level of experience with PSG*.
  a. In how many guideline projects have you used the PSG method?
  b. How did your level of experience with the method evolve over time?
2. Workflow Efficiency
*Objective: To understand how the method impacts the methodologist's workload and project flow*.
  a. Has the PSG method improved your workflow? In what ways?
  b. What changes, if any, have you observed in project timelines or communication processes?
3. Expert Panel Engagement
*Objective: To explore perceived changes in panelist involvement and team dynamics*.
  a. How did the PSG method affect panelist engagement and participation?
  b. Were there any differences in how panelists responded to this structure?
4. Lessons Learned for Other Methodologists
*Objective: To capture insights that could help peers adopt or refine the PSG method*.
  a. What are some key takeaways you would share with fellow methodologists?
  b. Are there any best practices you have developed when using this method?
5. Necessary Skillsets for Methodologists
*Objective: To identify core competencies required for methodologists using PSG*.
  a. What skills were especially important when implementing the PSG method?
6. Required Skills and Mindset for Panel Members
*Objective: To understand what makes the method work well from the panelists’ side*.
  a. What kinds of skills or attitudes do you believe panel members need for successful participation in PSG?

### 2.3 Data Extraction

Data extracted included the guideline title, number of panel experts, number of primary research questions (excluding sub‐questions), number of studies included in the systematic review, and the number of subgroups created for guidelines using the PSG method. The total duration of guideline development was calculated based on the core development phase, from the protocol approval date to the final approval date by the ASCO guideline approval body.

### 2.4 Statistical Analysis

#### 2.4.1 Quantitative Analysis

Descriptive statistics were used to summarize the characteristics and timelines of the guidelines developed under both the traditional and PSG method. Continuous variables, including guideline development time, number of panel members, research questions, and included studies, were summarized separately for each method using means and standard deviations.

The average duration and standard deviation (in months) from protocol approval to final committee approval was calculated for guidelines developed using each method, providing insight into typical timelines.

#### 2.4.2 Qualitative Analysis

A qualitative descriptive approach was used to analyze insights from ASCO guideline methodologists regarding their experiences with the PSG method. Data was collected through email correspondence and phone interviews. Phone interviews were transcribed verbatim, and all responses were compiled into a single data set. A thematic analysis approach was used to analyze the qualitative data. Initial coding was conducted by one researcher with expertise in guideline methodology and qualitative data analysis using an inductive approach, where recurring words, phrases, and ideas were tagged and categorized without imposing a predefined framework. A second coder independently reviewed the initial codes and themes. Codes and discrepancies were then reviewed and organized into broader themes through consensus discussions between the coders. NVivo 12 (QSR International) software was used to facilitate data organization, support coding consistency, and assist in the visualization of emergent themes. These themes were refined and organized into three major domains: (1) Perceived benefits, (2) Perceived challenges, and (3) Required skillsets. Subthemes were developed to capture more specific insights under each category. Illustrative quotes were selected to represent common or particularly insightful viewpoints within each thematic area. Descriptive information such as the number of guidelines developed using the PSG method and the range of years of experience of the respondents was summarized to provide context for the findings. The goal of the analysis was to explore shared experiences and reflections, rather than to quantify responses or test hypotheses.

### 2.5 Ethical Consideration

This study was conducted as an internal program evaluation within the ASCO Guidelines Department. Phase one involved a retrospective review of non‐identifiable administrative data related to guideline development. Phase two involved collecting insights from ASCO methodologists through structured questions delivered via email or phone interviews. No patient data were used, and no identifiable personal or sensitive information was collected. Participation in the second phase was voluntary and limited to ASCO staff engaged in guideline methodology work. As this study constituted an internal quality improvement initiative, formal ethics approval was not required.

### 2.6 Reporting Standards

This manuscript follows relevant reporting guidelines for each phase of the study. The retrospective review of administrative data (Phase 1) was reported in accordance with the *Strengthening the Reporting of Observational Studies in Epidemiology (STROBE)* checklist [10]. The qualitative component (Phase 2) was reported in line with the *Standards for Reporting Qualitative Research (SRQR)* checklist [11]. Completed checklists are included as supplementary materials.

## 3 Results

### 3.1 Results of Administrative Data Review

Since 2017, ASCO has published approximately 160 guideline products, with 16 meeting the inclusion criteria for this study. Seven guidelines were developed using the traditional method [12, 13, 14, 15, 16, 17, 18], while nine were developed using the PSG method [19, 20, 21, 22, 23, 24, 25, 26, 27]. The average number of panel members was 17 (SD, 2.67) for guidelines using the PSG method and 15 (SD, 2.05) for those using the traditional method. PSG‐developed guidelines included an average of 5 research questions (SD, 1.61), compared to 4 (SD, 1.03) in traditionally developed guidelines.

The average number of studies included was 142 (SD, 63.04) for the PSG group and 122 (SD, 67.34) for the traditional group. The average development time was 13 months (SD, 2.41) for PSG‐developed guidelines and 23 months (SD, 6.30) for guidelines developed using the traditional method. Among the PSG‐developed guidelines, the number of subgroups formed ranged from 2 to 6 (Tables 1 and 2).

**Table 1:** Guideline‐level data for traditional and PSG method groups.

Guidelines	Method	N of Months	N of Panel Members	N of Research Questions	N of Studies	N of Panel Subgroups
Malignant Pleural Mesothelioma [12]	Traditional	19	13	5	222	—
Management of Osteoporosis in Survivors of Adult Cancers [13]	Traditional	33	13	3	224	—
Exercise, Diet, and Weight Management During Cancer Treatment [14]	Traditional	23	13	3	75	—
Management of Metastatic Renal Clear Cell Cancer [15]	Traditional	19	16	5	54	—
Management of Fatigue in Adult Survivors of Cancer [16]	Traditional	32	17	2	113	—
Molecular Biomarkers in Localized Prostate Cancer [17]	Traditional	19	13	4	108	—
Therapy for Diffuse Astrocytic and Oligodendroglial Tumors in Adults [18]	Traditional	16	18	4	57	—
Treatment of Multiple Myeloma [19]	PSG	10	20	7	124	6
Management of Salivary Gland Malignancy [20]	PSG	14	16	6	293	6
Management of Stage III NSCLC [21]	PSG	16	16	5	127	5
Diagnosis and Management of SCC of Unknown Primary in the Head and Neck [22]	PSG	14	15	4	100	4
Systemic Therapy for SCLC [23]	PSG	12	16	8	95	4
Prevention and Management of ORN in Patients with Head and Neck Cancer [24]	PSG	10	21	6	80	5
Management of the Neck in SCC of the Oral Cavity and Oropharynx [25]	PSG	11	11	6	124	2
Integrative Oncology Care of Symptoms of Anxiety and Depression in Adults with Cancer [26]	PSG	16	17	4	110	4
Integrative Medicine for Pain Management in Oncology [27]	PSG	17	19	2	227	5

Abbreviations: N, number; NSCLC, non‐small cell lung cancer; ORN, osteoradionecrosis; PSG, panel subgroup; SCC, squamous cell carcinoma; SCLC, small cell lung cancer.

**Table 2:** Characteristics of guidelines developed using the traditional and PSG methods.

	Traditional method (*n* = 7)	PSG method (*n* = 9)
Characteristic	Mean (SD)	Mean (SD)
Number of panel members	15 (2.05)	17 (2.67)
Number of research questions	4 (1.03)	5 (1.61)
Number of studies included	122 (67.34)	142 (63.04)
Guideline development time (months)	23 (6.30)	13 (2.41)
Number of subgroups created	Not applicable	5 (1.11)

### 3.2 Results of Qualitative Interviews

#### 3.2.1 Uptake of the PSG Method

Six of eight ASCO guideline methodologists reported using the PSG method. Their experience ranged from working on 2 to 11 guidelines using this approach, with tenure at ASCO varying between 7 and 20 years. Data were gathered via email correspondence from five methodologists and through a phone interview with one.

### 3.3 Perceived Benefits

#### 3.3.1 Steady Workflow

Participants highlighted the PSG method's role in ensuring consistent progress in guideline development. Regularly scheduled subgroup meetings and frequency of communication between the methodologist and the panel minimized delays typically caused by waiting on individual panel responses. It also helped panelists become more familiar with the guideline content, enabling any member to represent the guideline in approval committee meetings as needed. Authorship justification was also ensured since this method enables contribution from all authors.

#### 3.3.2 Improved Panel Engagement

There was an overall perception that when panels were committed to the PSG process, the frequency of communication and organized subgroups meetings increased interactions between panel members and helped foster better relationships between panelists that sustained engagement throughout the guideline development process. These interactions facilitated more in‐depth discussions and review of evidence, ensuring all voices were heard.

#### 3.3.3 Shorter Development Time

Distributing tasks among subgroups was perceived to contribute to a shorter timeline for guideline development. This was attributed to the fact that the PSG method allowed for concurrent work across multiple sections in the guideline.

#### 3.3.4 Reduced Cochair Burden

Since the workload is shared between the working groups and panel members, co‐chairs are not overburdened with tasks enabling them to focus on synthesizing contributions.

### 3.4 Perceived Challenges

#### 3.4.1 Increased Time Commitment

Methodologists described the need for extensive administrative oversight, including managing email correspondence, attending meetings, and providing ongoing support to subgroups.

#### 3.4.2 Harmonizing Writing Styles

Due to the fact that the PSG method allows all panel members the opportunity to draft a section of the manuscript, the methodologists feel that this poses the challenge of integrating content from multiple authors, ensuring consistency and coherence in the final document.

#### 3.4.3 Panel Resistance and Commitment

Resistance to the PSG methods, particularly from co‐chairs or panelists unfamiliar with the process was identified as a barrier that can hinder successful implementation.

#### 3.4.4 Duplication of Efforts

There were instances where aspects of panel group work needed to be redone at the full panel meeting, this was attributed to overlap in research questions assigned to groups or gaps in subgroup expertise.

#### 3.4.5 Need for Oversight

The methodologists reported a need for constant oversight of the group work to ensure the groups are following instructions and completing tasks on time. They do also acknowledge that although this puts more demand on their time, the eventual payoff is worth the time spent.

#### 3.4.6 Burden on Select Experts

There was perception of more burden being put on some panel members with specific expertise particularly when their expertise is needed across multiple subgroups.

Direct quotes from participants that relate to themes on perceived benefits and challenges identified are included in Table 3.

**Table 3:** Participant quotes on perceived benefits and challenges when using the PSG method.

Perceived benefits	Perceived challenges
**Steady workflow** “I have used this method in many of the guidelines I have worked on, and I find that the regular communication helps keep the project moving and there are no long periods of silence between myself and the panel members. I become so familiar with the panel that it makes it easy for me to know when a panel member is not contributing to the process or manuscript.” (M1, used PSG in 11 guidelines) “It's usually a useful way to get draft recommendations for panel to consider in a way that is much more efficient for the panel to review them. Consensus is reached quicker.” (M6, used PSG in 6 guidelines)	**Increased time commitment** “The administration of subgroups has taken more time than working with Co‐Chairs alone (±another panel member). There are also increased communication responsibilities for both staff and Co‐Chairs which has require more meetings, phone calls, emails, reminders, etc…” (M2, used PSG in 6 guidelines) “It probably adds a bit of time on my end to coordinate and work with the various groups.” (M5, used PSG in 2 guidelines)
**Improved panel engagement** “Yes, I think it helped with engagement. Smaller groups can foster an environment of collaboration, and everyone has the chance to contribute.” (M4, used PSG in 3 guidelines) “I think that it has improved engagement by helping them feel more of a professional and substantive connection to the product.” (M3, used PSG in 2 guidelines)	**Harmonizing writing styles** “There are more volunteer emails and more text to integrate into the manuscript.” (M3, used PSG in 2 guidelines) “…and to reconcile the varying content that is produced.” (M5, used PSG in 2 guidelines)
**Shorter development time** “When I tried this on a guideline with a panel of about 24 members with multiple research questions, I had thought the guideline was going to take a longer time to complete but I was surprised at the duration it took which was shorter than expected. I believe this is due to the subgroup approach I took.” (M1, used PSG in 11 guidelines)	**Panel resistance and commitment** *“*When Co‐Chairs have been less receptive to PSGs it has been challenging to use this method.” (M2, used PSG in 6 guidelines) “Some subgroups deviated from the directions/methods and/or questioned the process and/or slowed down the process.” (M2, used PSG in 6 guidelines) “Some panels have been more receptive than others. A current guideline panel refused this approach. They thought it would be duplicative, repetitive, and inefficient.” (M4, used PSG in 3 guidelines)
**Reduced cochair burden** “It probably takes a bit of burden off of the co‐chairs but adds work for the other panel members.” (M5, used PSG in 2 guidelines)	**Duplication of efforts** “Many of the things the subgroups considered in their meetings/discussions came up again and had to be revisited in the full panel meetings.” (M4, used PSG in 3 guidelines) “It was also the case that some topics touched more than one subgroup, and this caused some redundancies.” (M4, used PSG in 3 guidelines)
	**Need for oversight.** “Without oversight, there will be inconsistencies in how the groups interpret/grade the evidence, deviations from the overall guideline goal, and a lack of overall cohesiveness.” (M4, used PSG in 3 guidelines) “It does generate more work for me. I would need to be on the call of the subgroups. The payoff in the long run is better.” (M6, used PSG in 6 guidelines)
	**Burden on select experts.** “Some experts need to participate in more than one group which creates more burden on some panel members.” (M4, used PSG in 3 guidelines)

#### 3.4.7 Required Skills

Overall, the methodologists feel that the same skills that are required for any method of engaging experts on a panel will be required for the PSG method. However, specific skills might be tested during this process or used more frequently. Some of these skills include:

- **Organization:** This was seen to be especially important for setting up efficient folder structures for the project and needed to manage ongoing email communication with panelists. Scheduling regular meetings, adapting them as needed, and using templates and collaborative tools (e.g., SharePoint, Outlook) to streamline project administration.
- **Communication:** The methodologists felt it was particularly important to have the skill to give clear instructions to the panel at each phase of the process. These include well‐crafted instructions given via email or during meetings and ongoing email correspondence. Setting clear timelines, regular progress updates, and reminders are essential, especially for clinicians with demanding schedules.
- **Time management:** This skill was said to be crucial to get the desired outcome of this process as methodologists are often managing multiple guideline products at any given point in time and experts on the panel usually have competing demands on their time. Therefore, this skill must be used efficiently to maximize productive engagement with panel members within the time they have available to spend on the guideline.
- **Editorial:** This skill is needed for harmonizing writing contributions from multiple authors. As most panel members might have different writing styles, the methodologists felt having strong editorial skills will make this step in the process less cumbersome. They also felt that giving clear instructions and providing templates or examples to panels can reduce the variation in writing styles.

The methodologists were also asked their perspectives on the skills and attitudes panel members need for successful PSG implementation. Their response was as follows:

- **Knowledge** ***:*** For successful implementation, the methodologists felt panel members need a clear understanding of the PSG method and a commitment to it. An early buy‐in from co‐chairs and group leads was important to promote commitment, accountability, and active participation of the panel.
- **Time commitment:** They also felt it was crucial for the experts to allocate time for regular meetings on their calendar and a commitment to completing assigned tasks promptly. Panel members also need to communicate with the methodologists promptly if they are unable to meet deadlines or no longer able to participate in the process as tardiness could cause a delay in the process.
- **Understanding and following instructions:** Understanding and following the guidance of the coordinating methodologist is important for a smooth workflow and to maintain consistency across all group work. This would also help reduce the burden of work on the co‐chairs and methodologists when they combine the group work in the manuscript.

**Figure 2:**
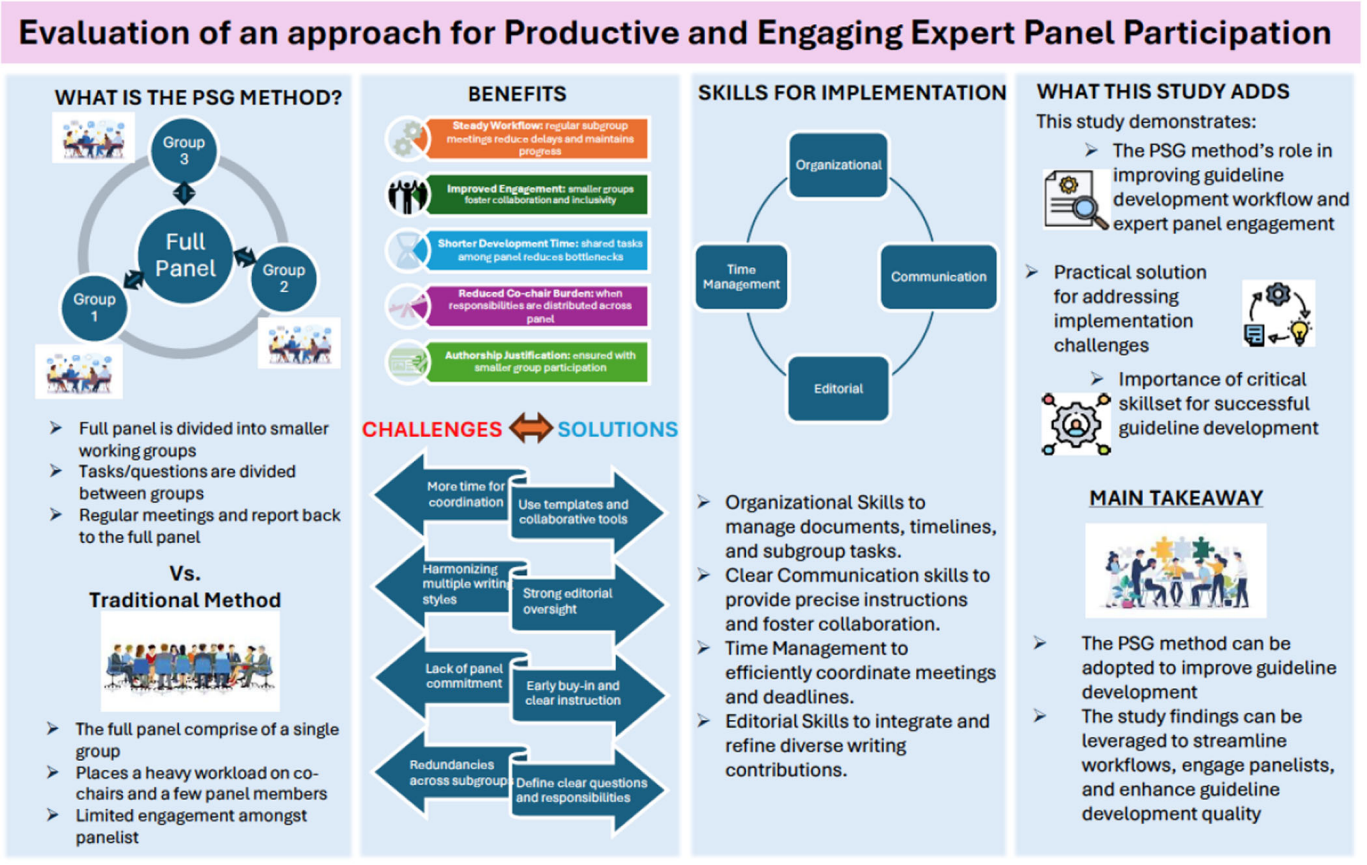
Infographic showing the assessment of the PSG method.

A visual summary of key findings is presented in the infographic (Figure 2).

## 4 Discussion

This study retrospectively reviewed administrative data from the ASCO guidelines department to examine expert panel engagement methods in guideline development. Our review showed that the PSG method led to faster guideline development, averaging 13 months, compared to the traditional method, which averaged 23 months. However, the perceptions of the methodologists from the qualitative interviews highlighted some challenges and benefits in implementing the PSG method. They reported extra time commitment, constant group oversight, harmonization of multiple writing styles, and duplicity of work in some instances as challenging while the method was thought to be beneficial in improving panel engagement and efficiency in guideline development. In addition, successful implementation of the PSG method required specific skills from both the expert panel members and the guideline methodologists including time management/commitment, communication, editorial, and organizational skills.

There are few studies done in this space, however, our findings align with a study by Borgonjen et al. in the Netherlands [6], which prospectively compared three guideline development methods for actinic keratosis treatment to evaluate whether alternative evidence‐based approaches would yield variations in clinical recommendations. Their Method 1 (standard multi‐session, evidence‐based approach) resembled our traditional method, while Method 2 (2‐day conference‐based formulation) was similar to our PSG method. Method 3 involved an epidemiologist summarizing evidence and a dermatologist drafting recommendations independently. The authors concluded that alternative, time‐efficient methods could be as valuable as the traditional approach for guidelines with a narrower scope and smaller evidence base. This alludes to the fact that despite differences in panel size, scope, and evidence volume, the Borgonjen study shows support of our findings which is that the traditional method appears to require significantly more time. Their study used smaller panels (seven members in Methods 1 and 2, and two members in Method 3) and fewer studies (approximately 60), yet Method 1 still took 14 months to complete, while Methods 2 and 3 required 5 and 4 months, respectively, which is a similar trend to our results.

### 4.1 Limitations

As a retrospective study, ours is subject to reporting and selection biases, as well as problems with incomplete data. The use of administrative data is also a limitation, as these databases are typically intended for administrative management rather than for research purposes, hence they may vary in detail and accuracy. Additionally, cost data, which could have provided valuable insights into the resource implications of these methods, was unavailable. Both the traditional and PSG methods were influenced by common variables, such as panel members' experience levels, response times, and variability in disease sites. The guidelines examined spanned multiple disease areas, adhering to panel composition guidelines outlined in the ASCO guidelines methodology manual [5]. A further limitation is that the lessons learned were drawn from the perspectives of six methodologists with varying length of experience using the PSG method. Feedback was not sought directly from expert panel members regarding their experiences with the PSG method. Instead, methodologists were asked to provide their views on the skills and mindsets they believed were necessary for successful panel member participation. The generalizability of our findings could be improved by expanding the pool of methodologists who have applied the PSG method and incorporating direct feedback from expert panel members both within ASCO and in other guideline development organizations. This would provide a more comprehensive understanding of the method and its reception among all stakeholders involved.

## 5 Conclusion

This study highlights the PSG method as a structured approach that may enhance productivity, engagement, and communication within guideline expert panels. Findings suggest that, compared to traditional methods, the PSG approach can streamline the guideline development process, even with larger panels and more complex scopes, resulting in shorter timelines. PSG's division of panel members into smaller, focused subgroups allowed for opportunities for deeper analysis of evidence, distributed workload, and more efficient decision‐making. It also encouraged consistent panel member engagement, supported authorship justification, and fostered closer networking within subgroups, which strengthened overall panel cohesion. In contrast, the traditional method places a disproportionate workload on the co‐chairs and staff, leaving the remaining panel members largely unengaged for extended periods while awaiting updates or tasks. This prolonged inactivity can result in disengagement and a sense of detachment from the project, potentially leading to diminished interest and commitment from panel members.

Despite the promising results, the PSG method requires essential skills from both panel members and guideline staff, including strong organizational, time management, and communication abilities, to maintain steady progress and adapt to panel dynamics. The challenges identified by the methodologists should also be taken into account when planning the application of this method. Given the variability of guideline topics, panel compositions, and research volume, the PSG approach may be best suited for guidelines with substantial evidence bases or numerous research questions. This method's effectiveness may vary, so guideline development teams should consider panel size, expertise distribution, and project timelines when determining the best approach.

In summary, the PSG method provides a feasible alternative to traditional methods, potentially offering a more effective and efficient way to complete guideline development. It has the potential to be particularly beneficial for organizations aiming to produce high‐quality guidelines within shorter timelines, fostering improved engagement, satisfaction, and contribution from expert volunteers.

## Author Contributions

**Nofisat Ismaila:** conceptualization, data curation, formal analysis, investigation, methodology, project administration, resources, software, visualization, writing – original draft, writing – review and editing. **Brittany E. Harvey:** project administration, writing – review and copyediting. **Kaitlin Einhaus:** project administration, writing – review and editing. **Lawrence Mbuagbaw:** supervision validation, writing – review and editing. **Jinhui Ma:** supervision validation, writing – review and editing. **Lehana Thabane:** supervision validation, writing – review and editing.

## Ethics Statement

This study involved a retrospective review of administrative data and qualitative feedback from ASCO staff members. As the study was conducted entirely within the organization and did not involve human subjects research, it was deemed exempt from institutional review board oversight.

## Conflicts of Interest

The authors declare no conflicts of interest.

## Data Availability

The data supporting the findings of this study are available from the corresponding author upon request. Administrative data were obtained from internal ASCO records, and qualitative responses were collected from ASCO staff members.
